# Evaluation of Paraoxonase-1 and Pentraxin-3 in the Diagnosis and Prognosis of Endometrial Cancer

**DOI:** 10.3390/antiox11102024

**Published:** 2022-10-13

**Authors:** Mateusz Kozłowski, Kaja Michalczyk, Grzegorz Witczak, Sebastian Kwiatkowski, Aneta Mirecka, Katarzyna Nowak, Ewa Pius-Sadowska, Bogusław Machaliński, Aneta Cymbaluk-Płoska

**Affiliations:** 1 Department of Gynecological Surgery and Gynecological Oncology of Adults and Adolescents, Pomeranian Medical University in Szczecin, al. Powstańców Wielkopolskich 72, 70-111 Szczecin, Poland; 2 Department of Obstetrics and Gynecology, Pomeranian Medical University in Szczecin, al. Powstańców Wielkopolskich 72, 70-111 Szczecin, Poland; 3 Department of General Pathology, Pomeranian Medical University in Szczecin, al. Powstańców Wielkopolskich 72, 70-111 Szczecin, Poland

**Keywords:** paraoxonase-1, pentraxin-3, PON1, PTX3, diagnostic marker, prognostic factor, endometrial cancer

## Abstract

It is relevant to find new prognostic and diagnostic biomarkers for endometrial cancer. The study group consisted of 94 cases of endometrial cancer, the control group of 65 cases of normal endometrium. We evaluated PON1 and PTX3 serum levels. The ROC curve was plotted. The area under the curve was calculated to characterize the sensitivity and specificity of the studied parameters. Univariate and multivariate analyses were performed simultaneously using the Cox regression model. The Kaplan–Meier curve was used to assess survival. The cut-off level of PON1 was 142.6 ng/mL, with a sensitivity and specificity of 79 and 84% (*p* = 0.0321). The cut-off level of PTX3 was 4.2 ng/mL, with a sensitivity and specificity of 63 and 57% (*p* = 0.028). The favorable prognostic factor determined in serum was PON1 (for PFS: HR 0.93, 95% CI 0.86–1.03, *p* = 0.046; for OS: HR 0.96, 95% CI 0.89–1.08, *p* = 0.009). PON1 may be considered a potential biomarker in the diagnosis of endometrial cancer. Considering multivariate analysis, the PON1 serum level above the median is an independent favourable prognostic factor affecting PFS and OS. Considering Kaplan–Meier curves, longer recurrence-free survival and overall survival were found in patients with PON1 levels below the median. In view of the inconclusive results, we suggest that further studies should be conducted.

## 1. Introduction

Endometrial cancer is recognized as one of the most common cancers in women. Its incidence is still increasing, not only in Western Europe but also in Central and Eastern Europe [1]. The pathogenesis of endometrial cancer is multifactorial, and known risk factors include age, race, metabolic syndrome, unopposed estrogen exposure and genetic predispositions [2]. However, it is important to note the significant role of oxidative stress in the development of many metabolic diseases. As it turns out, however, oxidative stress also plays a role in the carcinogenesis of many malignancies, which has been the subject of research for several years. Oxidation and reduction reactions lead to the formation of damaging molecules that affect cells. Imbalances between the production and accumulation of reactive metabolites and free radicals lead to oxidative stress. A key mechanism leading to oxidative damage is the hydroxyl radical. Other mechanisms that cause the modification of nucleic acids may be due to the action of lipid peroxidation products capable of forming adducts with DNA [3]. Oxidative stress leads to the adherence of monocytes to the vascular endothelium through the activation of lipid peroxidation products, which causes the synthesis of pro-inflammatory factors [4]. The expression of pentraxin-3 (PTX3) in endothelial cells can be regulated by a number of cytokines. PTX3 belongs to the pentraxin family, which is produced as response to inflammatory mediators and has numerous functions in different physiopathological conditions, including cancer [5,6]. PTX3 is engaged in tumor cell proliferation, angiogenesis, metastasis and immune modulation in tumors. Current reports show that PTX3 is a product of both neoplastic and stromal cells, changing the tumor microenvironment [7]. Some publications report that PTX3 may also act as an oncosuppressor by modulating tumor-associated inflammation or by blocking tumor growth factors such as different members of the FGF family [8,9].

Paraoxonase-1 (PON1) has exactly the opposite effect. Paraoxonase-1 acts as an antioxidant. Its protective role in inhibiting lipid peroxidation products is being increasingly publicized. PON1, as a lipolactonase, is involved in scavenging mechanisms and in the elimination of carcinogenic free radicals in order to maintain the oxidative balance [10]. Moreover, the latest research shows that the amount of paraoxonase-1 in patients with diabetes and metabolic syndrome may be reduced, which would correlate with its deficit in patients with endometrial cancer [11,12].

The aim of the study was to determine the clinical relevance of the tested proteins. We evaluated paraoxonase-1 and pentraxin-3 in the diagnosis and prognosis of endometrial cancer.

## 2. Materials and Methods

### 2.1. Study Design

The study involved patients who underwent abrasion due to perimenopausal bleeding. Subsequently, patients with a histopathological diagnosis of endometrial cancer were qualified for oncological surgery. A study group with endometrial cancer and a non-cancer control group were distinguished. The study group was then divided into subgroups considering histological subtype, staging, grading, lymphnode metastasis and lymphovascular space invasion. Serum levels of PON1 and PTX3 were determined in both the study and control groups to assess their significance as diagnostic markers and prognostic factors in endometrial cancer.

### 2.2. Participants

The study initially included 169 patients treated in the Department of Gynecological Surgery and Gynecological Oncology of Adults and Adolescents for perimenopausal uterine bleeding. The exclusion criteria for the study were lack of patient consent, history of treatment for another cancer, pelvic inflammatory disease, incomplete patient data, histological diagnosis of uterine malignancy other than cancer, unbalanced chronic diseases, autoimmune diseases. After the results of the histopathological examination, 4 patients with endometrial carcinosarcoma and 2 patients with endometrial sarcoma were excluded from the study in order to maintain the homogeneity of the group. Moreover, 2 patients with unbalanced diabetes were excluded from the study, as the course of the disease could have potentially affected serum concentrations of PON1. Additonally, 1 patient with rheumatoid arthritis and dermatomyositis was excluded, as the primary diseases might have distorted the values of serum PON1 and PTX3. Finally, 159 patients were included in the study.

At the beginning of the study, the patients’ body mass index (BMI) was measured based on the patients’ weight and height. The BMI was calculated based on the formula: BMI = weight [kg]/height^2^ [m^2^]. Based on the results, the patients were qualified into three subgroups: with normal weight (BMI 18.5–24.9), overweight and obese (BMI > 30). Moreover, each of the patients had a blood pressure measurement. Based on the results, we divided the patients into a group with (>140/90) and without hypertension. Moreover, based on the patients’ medical history, we assessed the presence of type 2 diabetes (DM2). During the admission of the patient to the hospital, we have also performed routine blood measurements. One of them was serum CRP (C-reactive protein), which is a protein whose levels may be influenced by the proinflammatory action of the cytokines secreted by the adipose tissue. Based on the results (below and above standard cut-off value), we divided the patients into two subgroups. The group characteristics are listed in Table 1 and Table 2.

### 2.3. Treatment Regimens

The patients who participated in the study underwent one of the following procedures: dilation and curettage (D&C), hysteroscopy or surgery. In our study, we divided the patients based on the histopathological result (into patients with benign vs. malignant lesions and then into endometrial and non-endometrial endometrioid carcinoma). In case of a histopathological diagnosis of a malignancy, patients underwent surgery. The surgical procedure depended on staging and was based on European Society of Gynaecological Oncology (EGSO) guidelines. Patients diagnosed with benign gynecological lesions were qualified into the controlled group.

### 2.4. Analysis of Serum Biomarkers

After obtaining consent from the patient, an additional sample of 5 mL of whole blood was collected from the patient at the preoperative stage. The blood was centrifuged. Then the obtained serum was stored at −70 °C for further examination. We used multiplex fluorescent bead-based immunoassays (Luminex Corporation, Austin, TX, USA) and commercial Bio-Plex Pro RBM Human Metabolic Panel 2 #171AMR2CK (Bio-Rad, Hercules, CA, USA) to measure serum PON1 and PTX3 concentrations. During the test, we added 30 µL of standard, control and action on the plate. Then, we added 10 µL of blocker with 10 µL downstream of the antibody capture multiplex to all wells. We incubated the plate for one hour at room temperature. After the incubation time, we individually washed each well three times using the test buffer. Next, 40 µL of antibody detection cocktail was added to each well. Then, the plate was placed on a shaker and incubated for 1 h at room temperature, followed by the addition of a streptavidin-phycoerythrin mixture to the plate. The samples were incubated once more with stirring for 30 min in the dark. Having washed the microspheres in each well, we added the assay buffer and shaked the probes for 30 s at room temperature. The plates were then analyzed using Luminex technology.

### 2.5. Statistical Analysis

The statistical calculations were made using Statistica 10 software (TIBCO Software Inc.). Descriptive analysis was used to characterize the examined group of patients. The distribution of data in the studied group of patients was not normal and not homogeneous. Therefore, we performed the analysis using non-parametric tests. To compare the values between two group, the U Mann–Whitney test was used. Kruskal–Wallis test and Dunn’s post-hoc tests were used to compare the values between three groups. Moreover, as a part of the analysis, we used Spearman’s rank correlation coefficient. We used receiver operating characteristics (ROCs) to assess the combined sensitivity and specificity of the tested parameters. We performed univariant and multivariant logistic regression models using Cox regression to assess the HRs and the 95% CIs for the associations between patients’ risk factors for endometrial cancer and studied protein concentrations. The analyzed parameters included patients’ age, FIGO stage, tumor grade, menopausal status, BMI, CRP level and tested biomarker. The considered indicator of statistical significance was *p*-value < 0.05.

## 3. Results

### 3.1. Comparison of Serum Concentrations of Tested Biomarkers in Groups and Subgroups

We found statistically significant differences in the median serum levels for PON1 and PTX3 in the group of endometrial cancer, when compared to the group of normal endometrium (without taking into account the hormonal status of the patients). In case of postmenopausal patients, statistically significantly lower median PON1 levels were noted in patients with endometrial cancer compared to the control group (*p* = 0.002). We found no significant differences in serum concentrations for PTX3. In the group of premenopausal patients, we found significantly lower median concentrations of PON1 in the group of patients with endometrial cancer as compared to the group with normal endometrium (*p* = 0.01). We also found statistically significantly higher median concentrations for PTX3 (*p* = 0.04). We found no statistically significant differences in the median age between the study group and the control group (57.9 vs. 54.2 years). Moreover, there were no statistically significant differences in the median BMI. In the study, we noticed a greater number of patients with DM 2 and HA in the group of patients with endometrial cancer compared to the control group (*p* = 0.031 and *p* = 0.084, respectively). The results are shown in Table 1, Table 2 and Table 3.

When comparing concentrations across grading subgroups, PON1 levels were found to be statistically significantly higher in G1 cancers in premenopausal patients (*p* = 0.043). The remaining differences in concentrations were non-significant. Concentrations in the staging subgroups were also compared. Significant differences were found in PON1 and PTX3 concentrations in the groups of patients, regardless of menopausal status (*p* = 0.022, *p* = 0.041, respectively). In the group of premenopausal patients, significantly higher PON1 levels were found in patients with staging I-II compared to III-IV (*p* = 0.031). Differences for PTX3 were not statistically significant. In postmenopausal patients, significantly higher PON1 levels were found in patients with staging I-II compared to III-IV (*p* = 0.009). These patients also had significantly lower PTX3 levels (*p* = 0.003). The exact results are shown in Table 4 and Table 5.

### 3.2. Correlations between Studied Variables

**Figure 1:**
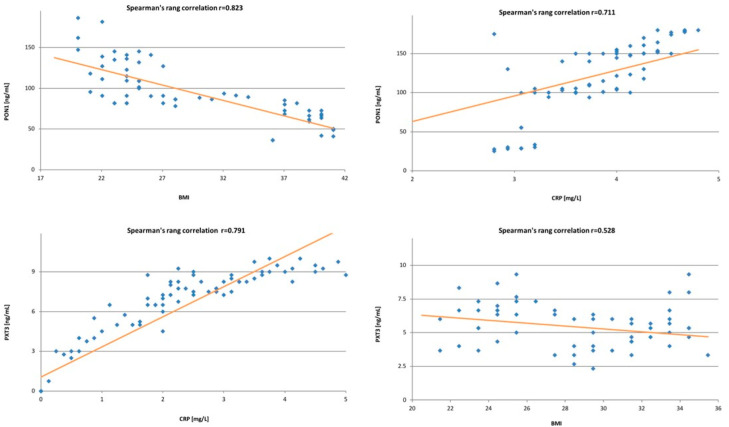
Spearman’s correlations between the levels of studied proteins and patients’ BMI and CRP levels. On the (**top**), figures demonstrate correlations for PON1, while on the (**bottom**), figures demonstrate correlations for PTX3.

Due to the abnormal distribution of the group, we performed an analysis to see whether there was a relationship between the tested markers using a non-parametric Spearman correlation coefficient. In the study, we found no correlation between paraoxonase-1 and pentraxin-3. Moreover, we performed an analysis assessing the impact of individual risk factors for endometrial cancer on protein levels. We found a strong correlation between paroxonase-1 and patients’ BMI (rs = 0.823; *p* = 0.003) and a weak correlation between paraoxonase-1 and type 2 diabetes (rs = 0.608; *p* = 0.02). However, we did not notice such correlations between DM2 and pentraxin-3. In our study, we found that PTX3 has a weak negative correlation with patients’ BMI (r = 0.528). Moreover, we demonstrated strong correlations between PON1, PTX3 and the acute phase protein CRP ( rs = 0.711; *p* = 0.004, rs = 791; *p* = 0.01, respectively) (Figure 1).

### 3.3. Receiver Operating Characteristic (ROC) Curve for Using PON1 and PTX3 Distinguishing between Endometrial Cancer and Normal Endometrium

**Figure 2:**
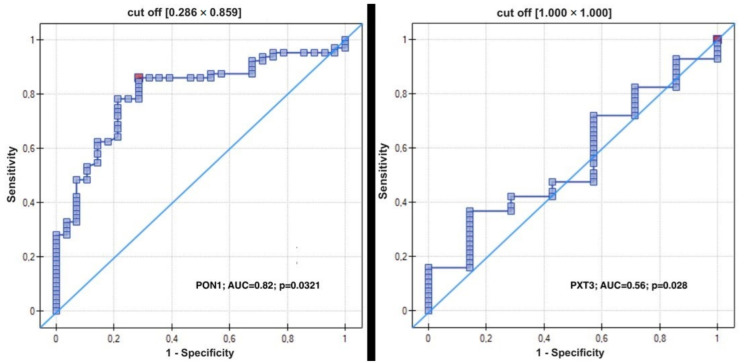
(ROC) Curve using PON1 (**left**) and PTX3 (**right**) Distinguishing between endometrial cancer and normal endometrium.

The cutoff values for PON1 and PTX3 that were elevated in the serum of patients were calculated using ROC curve analysis. The analysis showed that when the serum PON1 level was 142.6 ng/mL or higher, the sensitivity and specificity were 79 and 84%, respectively (*p* = 0.0321). When the serum PTX3 level was 4.2 ng/mL or higher, the sensitivity was 63% and specificity was 57% (*p* = 0.028) (Table 6). The area under the curve (AUC) for PON1 was 0.82 and for PTX3 was 0.56. The ROC curves for PON1 and PTX3 are shown in Figure 2.

### 3.4. Survival Analysis Using COX Regression

#### 3.4.1. COX Regression for PON1

In univariate analysis, the length of progression-free survival (PFS) was influenced by staging, BMI and preoperative serum level of PON1 (*p* = 0.01, *p* = 0.008, *p* = 0.033, respectively). We should point out that only in the PON1 level was the HR < 1 (0.89). Ooverall survival (OS) was influenced by age (*p* = 0.049), staging (*p* = 0.03), BMI (*p* = 0.047) and level of PON1 (*p* = 0.016). The HR for PON1 was 0.88. In multivariate analysis, the independent risk factor influencing OS was staging (*p* = 0.001). PON1 levels were an independent protective factor (HR = 0.96, *p* = 0.009). In turn, independent factors influencing PFS were age (*p* = 0.044), staging (*p* = 0.002), BMI (*p* = 0.048), CRP level (*p* = 0.008) and PON1 concentration (HR = 0.93, *p* = 0.046). A presentation of the effect of the risk factors studied and the PON1 level is shown in Table 7.

#### 3.4.2. COX Regression for PTX3

In univariate analysis, the length of PFS was influenced by age (*p* = 0.048), staging (*p* = 0.013), grading (*p* = 0.022) and preoperative serum concentration of PTX3 (*p* = 0.043). OS was affected by staging (*p* = 0.021), CRP level (*p* = 0.043) and PTX3 level (*p* = 0.013). In multivariate analysis, independent risk factors influencing OS were staging (*p* = 0.003) and CRP level (*p* = 0.049). HR for PTX3 level was 1.31 but the result was non-significant (*p* = 0.321). In turn, an independent factor influencing PFS was staging (*p* = 0.039). HR for PTX3 level was 1.24, but the result was non-significant (*p* = 0.134). A presentation of the effect of the studied risk factors and the PTX3 level is shown in Table 8.

### 3.5. Survival Analysis Using the Kaplan–Meier Curves

**Figure 3:**
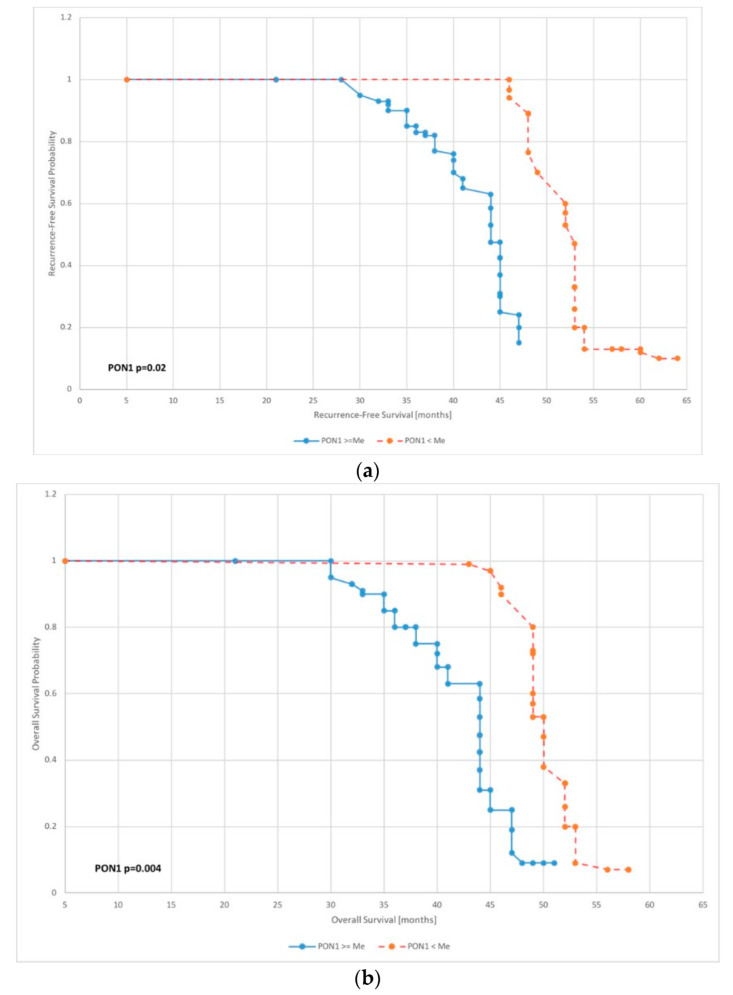
Kaplan–Meier recurrence-free survival curve (**a**) and overall survival curve (**b**) for endometrial cancer patients based on serum PON 1 level.

A survival analysis achieved by applying the Kaplan–Meier curve was conducted to define the utility of the proteins studied in the survival prognosis of endometrial cancer. Overall survival (OS) analysis and recurrence-free survival (RFS) analysis using Kaplan–Meier curves were conducted for PON1 median levels. RFS probability and OS probability were found to be higher for PON1 concentrations below the median (*p* = 0.02, *p* = 0.004, respectively) (Figure 3).

## 4. Discussion

Endometrial cancer is a hormone-dependent malignancy. In 80% of cases, this concerns obese females, diagnosed with metabolic syndrome [13]. In the last decade or so, excessive, unhealthy nutrition, mainly based on fast food, has prevailed in many countries, with a simultaneous sedentary lifestyle without sufficient physical activity, resulting in a spike in the diagnosis of metabolic disorders [14]. There is still ongoing research looking for the endogenous factors that are present in endocrine, pro-inflammatory environment, and under oxidative stress, these could serve as sensitive and specific determinants linking metabolic disorders with carcinogenesis processes. In our research, we tested two proteins, paraoxonase-1 and pentraxin-3. Both proteins are closely related to inflammation, cytokines and the oxidative stress that occurs in obese patients in the adipose tissue, especially visceral fat, which acts as an endocrine organ. PON1 belongs to a group of enzyme proteins that also includes paraoxonase-2 (PON2) and paraoxonase-3 (PON3). Paraoxonases exhibit broad enzymatic activity against a variety of substrates. Paraoxonase-2 demonstrates lactonase and very low arylesterase activity. In contrast, paraoxonase-3 shows high lactonase activity, weak arylesterase activity and almost no paraoxonase activity. Paraoxonase-1, on the other hand, shows all three activities [15]. As previous studies have shown, it is obvious that PON1 plays a role in cancers. The variability of PON1 activity in malignancies is extensive. Low PON1 activity enhances oxidative stress, suggesting a worse prognosis in patients with malignancies [16]. In addition, PON1 polymorphisms and their association with various cancers have also been studied, although more research showing the importance of PON1 polymorphisms in cancer development is required [17,18]. In our study, we found a negative correlation between the PON1 and patients’ BMI. The same results were demonstrated by Asman, Mehmet et al. [19], Ferretti et al. [20] and Bajnok et al. [21]. In the current literature, there is only one publication by Rector et al. [22], showing lower PON1 concentrations in patients with weight loss. The results are slightly different for PTX3. In our study, we found a weak negative correlation between BMI and PTX3. A similar correlation was described in a study by Witasp et al. [23], who found that the negative correlation was stronger in patients who were not overweight nor obese. Moreover, in the group of patients with weight loss, an increase in PTX3 was found, which confirmed the dependence between PTX3 levels and body weight. PON1 and PTX3 are not only related to body mass, but also play an active part in the regulation of carbohydrate and lipid metabolism. However, PON1 counteracts insulin resistance, not using the insulin receptor, but acting through an enhanced expression of the PI3K/Akt signaling pathway. This pathway promotes the translocation of GLUT4 vesicles to the cell membrane, which results in an increased glucose uptake [24]. Moreover, PON1 is closely related to high-density lipoproteins (HDL), and prevents the accumulation of lipid peroxides in low-density lipoproteins (LDL) [25]. In our study, we found a correlation between PON1 and type 2 diabetes. In previous research, Lettelier [26] and Mackness et al. [27] found that hyperinsulinemia, dyslipidemia and oxidative stress lead to a decrease in serum PON1 levels [26,27,28].

In our study, in contrast to previous reports, we did not find any correlation between PTX3 and type 2 diabetes. Takashi et al. [29] described a positive correlation of PTX3 with both hyperglycaemia and type 2 diabetes. Moreover, there are studies suggesting a correlation between increased PTX3 concentration in patients and insulin resistance in patients with PCO syndrome [30]. Obesity is known to be accompanied by chronic systemic inflammation, characterized by macrophage infiltration, particularly in visceral tissue, increased levels of inflammatory substances in the plasma, and excessive lipid accumulation. In our study, we found a positive correlation between both PTX3, PON1 and C-reactive protein (CRP). It should be noted that pentraxin belongs to the same group of acute phase proteins as CRP. However, in contrast to the production of CRP, which takes place in the liver, its production is only peripheral [31,32].

In our study, we noted a strong relationship between PON1, PTX3 and endometrial cancer. We noted statistically lower levels of PON1 in patients with endometrial cancer, even accounting for a potential risk factor for endometrial cancer, which is body mass index, by including this in the control group patients of similar BMI. Similar results were presented by Arioz et al. [33] in their publication, where they found significantly lower serum concentrations of PON1 in the group of patients with endometrium cancer. Our research confirms the previous findings on a bigger group of patients. The results seem to be valid and justified by the presumed role of PON1 as an antioxidant factor, as tumor growth and metastasis are closely related to oxidative stress and inflammatory processes. In the absence of PON1 in the microenvironment, chronic inflammation and oxidative stress lead to cell damage and, in the next stage, to carcinogenesis [34,35,36]. Moreover, there are potentially carcinogenic compounds, such as 8-oxo-deoxoguanisine, which accumulate in the absence of lipid peroxidation by PON1. To date, there have been limited publications reporting the serum PON1 concentrations, activity, or expression in patients with endometrial cancer. Gałczyński et al. [37] have proved that PON1 activity against paraoxone and phenyl acetate was statistically significantly decreased in patients with endometrial carcinoma. We found no previous reports on the possible use of PON1 as a prognostic factor, but our research seems very promising.

A study by Samra et al. [38] showed that the decreased level of paraoxonase-1 was present in different types of malignancies, including breast cancer, prostate cancer, non-Hodgkin and Hodgkin’s lymphoma. Unfortunately, the size of the study groups was so small that the results cannot be treated unequivocally. Considering subsequent cancers individually, Eroglu et al. [39] found elevated levels of PON1 in patients with prostate cancer. Similarly, Asfar et al. [40] demonstrated higher serum PON1 concentrations in patients with colorectal cancer, when compared to the control group. On the other hand, Elikran et al. [41] proved that serum PON1 concentrations were significantly lower in patients with lung cancer. The different results obtained by various researchers suggest the need for further studies to establish the actual role of PON1 in different types of cancer.

However, it should be noted that the results presented in this study are inconclusive. Both multivariate analysis and survival assessment presented by the Kaplan–Meier curve were used to assess prognostic significance. Multivariate analysis showed that PON1 levels above the median were an independent favorable prognostic factor for both PFS and OS. In contrast, the Kaplan–Meier curve showed that longer recurrence-free survival and overall survival were found in patients with PON1 levels below the median. Yu et al. showed that the downregulation of PON1 suggests a higher recurrence rate in patients with hepatocellular carcinoma [42]. Nevertheless, there are still few data from the literature describing the prognostic significance of PON1 in cancers, especially gynecological cancers. The results presented in this paper point to PON1 as a significant prognostic factor in endometrial cancer. However, they also indicate the need for further studies to accurately determine the prognostic significance of PON1.

Many researchers agree that the role of PTX3 in cancer has not been yet fully established. Pentraxin-3 appears to play a double role in cancer. On one hand, its overexpression is being described as an unfavorable prognostic marker in some types of neoplasms. On the other hand, its anti-angiogenic effect and antitumor properties may bear signs of oncosuppression manifested in other types of tumors. The researchers emphasize that the effect of PTX3 will be closely dependent on the type of tumor and its microenvironment. In our study, we found statistically significant higher levels of PTX3 in patients with endometrial cancer compared to the control group. Unfortunately, there are no previous reports to which we could compare our results of PTX3 levels in patients with endometrial cancer. However, it was previously found on several in vitro cell lines that the overexpression of PTX3 promotes migration and facilitates the invasion of malignant cells into linear pancreatic cells [43]. Other studies have also shown that the expression of PTX3 is elevated in gastric cancer tissues and induces tumor-associated gastritis by increasing the migration of macrophages and neoplastic cells [44]. In vitro studies of cervical cancer revealed a correlation between the expression of PTX3 and the differentiation of this cancer. A very promising study of pentraxin-3 on patients with small cell lung cancer found an increased expression of PTX3 in malignant tissues. Moreover, its expression was closely correlated with a shorter disease-free and shorter overall survival time of these patients. Considering the sensitivity and specificity of PTX3 (63% and 57%, respectively), we do not suggest using this marker in the diagnosis of endometrial cancer. The multivariate analysis that was performed did not demonstrate the significance of this biomarker in the prognosis of endometrial cancer. Nevertheless, univariate analysis indicated that PTX3 influences PFS and OS, so we suggest that further research on this protein should be pursued, especially since there are few such data in the literature.

## 5. Conclusions

Paraxonase-1 is a clinically relevant biomarker in endometrial cancer. PON1 could be used as a marker in the diagnosis of endometrial cancer. Pentraxin-3, due to its relatively low sensitivity and specificity, requires further study. Considering multivariate analysis, a PON1 serum level above the median is an independent favourable prognostic factor affecting PFS and OS. Considering Kaplan–Meier curves, longer recurrence-free survival and overall survival were found in patients with PON1 levels below the median. In view of the inconclusive results, we suggest that further studies should be conducted.

## Figures and Tables

**Table 1:** Characteristics of patients including endometrial cancer characteristics.

Characteristics	Number of Patients (%)
Endometrial cancer	
Yes	94 (59)
No	65 (41)
Endometrial Cancer	
Endometrial endometrioid carcinoma	81 (86)
Non-endometrial endometrioid carcinoma	13 (14)
Clinical staging	
FIGO I and II	66 (70)
FIGO III and IV	28 (30)
Histopathological grading	
Grade 1	34 (36)
Grade 2	39 (41)
Grade 3	21 (22)
Lymphnode metastasis	
Yes	28 (30)
No	66 (70)
Lymphovascular space invasion (LVSI)	
Yes	51 (54)
No	43 (46)

**Table 2:** Characteristics of patients including clinical and demographic data.

Clinic-Demographic Characteristics	Total Cohort (*n* = 159)	Endometrial Cancer (*n* = 94)	Normal Endometrium (*n* = 65)	*p*-Value
Median (IQR)				
Age (years old)	56.1 (45.2–66.8)	57.9 (47.21–71.8)	54.2 (43.1–55.2)	0.058
BMI (kg/m^2^)	26.1 (24.9–29.9)	28.4 (24.1–32.7)	25.9 (22.3–28.2)	0.121
Number (%)				
Age (years old)				
<65	88 (55)	39 (41)	39 (60)	0.062
≥65	71 (45)	55 (59)	26 (40)	0.048
BMI				
<25	30 (19)	14 (15)	16 (25)	0.213
≥25	129 (81)	80 (85)	49 (75)	0.041
Menopausal status				
Premenopausal	68 (43)	33 (35)	35 (54)	0.687
Postmenopausal	91 (57)	61 (65)	30 (46)	0.003
Hypertension				
Yes	111 (70)	67 (71)	44 (68)	0.084
No	48 (30)	27 (29)	21 (32)	0.583
Type 2 diabetes				
Yes	106 (67)	67 (71)	39 (60)	0.031
No	53 (33)	27 (29)	26 (40)	0.823
CRP level				
<5	96 (60)	61 (65)	34 (52)	0.049
≥5	63 (40)	33 (35)	31 (48)	0.466

**Table 3:** Comparison of PON1 and PTX3 levels between patients with endometrial carcinoma and the control group.

Characteristics		Endometrial Cancer	Normal Endometrium	*p*-Value
PON1 [ng/mL]	Median	136.2	173.4	0.006
	IQR	(119.1–151.4)	(159.5–181.7)	
PON1 [ng/mL] premenopausal	Median	129.8	171.6	0.01
	IQR	125.4–137.3)	(156.3–180.4)	
PON1 [ng/mL] postmenopausal	Median	131.7	164.6	0.002
	IQR	115.1–140.7	158.9–176.2	
PTX3 [ng/mL]	Median	8.1	3.9	0.0036
	IQR	7.2–9.5	3.3–4.8	
PTX3 [ng/mL] premenopausal	Median	7.5	4.3	0.040
	IQR	6.6–8.8	4.1–5.3	
PTX3 [ng/mL] postmenopausal	Median	8.4	7.0	0.108
	IQR	7.8–10.4	6.2–8.1	

**Table 4:** Comparison of PON1 and PXT3 levels between patients with G1 and G2-G3 endometrial carcinoma.

Characteristics		G1	G2–G3	*p*-Value
PON1 [ng/mL]	Median	163.4	148.7	0.094
	IQR	120.4–189.9	112–159.2	
PON1 [ng/mL] premenopausal	Median	166.2	140.1	0.043
	IQR	129.8–184.1	117.5–160.6	
PON1 [ng/mL] postmenopausal	Median	159.1	143.6	0.219
	IQR	144.2–171.1	129.7–157.5	
PTX3 [ng/mL]	Median	5.8	7.5	0.307
	IQR	3.7–6.9	6.3–8.8	
PTX3 [ng/mL] premenopausal	Median	5.5	7.3	0.129
	IQR	4.4–6.9	5.9–8.2	
PTX3 [ng/mL] postmenopausal	Median	6.1	8.0	0.051
	IQR	4.2–7.4	5.5–8.8	

**Table 5:** Comparison of PON1 and PXT3 levels between patients with stage I–II and stage III–IV endometrial carcinoma.

Characteristics		Stage I–II	Stage III–IV	*p*-Value
PON1 [ng/mL]	Median	184.2	145.5	0.022
	IQR	152.7–189.9	126.2–161.6	
PON1 [ng/mL] premenopausal	Median	173.4	147.9	0.031
	IQR	133.4–189.8	120.6–159.5	
PON1 [ng/mL] postmenopausal	Median	184.8	142.1	0.009
	IQR	149.6–190.2	128.8–158.7	
PTX3 [ng/mL]	Median	4.9	7.9	0.041
	IQR	3.9–5.6	6.1–8.8	
PTX3 [ng/mL] premenopausal	Median	5.5	7.6	0.063
	IQR	4.2–6.9	6.0–8.2	
PTX3 [ng/mL] postmenopausal	Median	4.6	8.1	0.003
	IQR	4.2–59	6.7–8.8	

**Table 6:** The diagnostic values of PON1 and PTX3 for patients with endometrial cancer.

Marker	AUC (95% CI)	Sensitivity [%]	Specificity [%]	*p*-Value	Cut-Off Value [ng/mL]
PON1	0.82	79	84	0.0321	142.6
PTX3	0.56	63	57	0.028	4.2

**Table 7:** Univariate and multivariate Cox regression model for PON1 serum concentrations.

Univariate Analysis						
Variable	PFS			OS		
	HR	95% CI	*p*-Value	HR	95% CI	*p*-Value
Age (above vs. below median)	1.28	0.63–1.14	0.102	1.09	0.92–1.121	0.049
FIGO staging (III and IV vs. I and II)	1.98	1.42–2.08	0.010	1.79	1.49–2.07	0.030
Grade (G3 vs. G1)	1.31	1.20–1.56	0.084	1.12	0.90–1.31	0.213
Menopausal status (postmenopausal vs. premenopausal)	1.18	1.09–1.29	0.063	1.11	1.03–1.21	0.059
BMI (≥25 vs. <25)	1.21	1.13–1.26	0.008	1.25	1.19–1.27	0.047
CRP level (≥5 vs. <5)	1.12	1.07–1.16	0.087	1.18	1.15–1.20	0.069
PON1 level (above vs. below median)	0.89	0.76–1.02	0.033	0.88	0.87–1.28	0.016
**Multivariate Analysis**						
	**PFS**			**OS**		
	**HR**	**95% CI**	***p*** **-Value**	**HR**	**95%CI**	***p*** **-Value**
Age (above vs. below median)	1.06	0.91–1.09	0.044	1.14	1.07–1.21	0.069
Stage (III and IV vs. I and II)	1.43	1.39–1.56	0.002	1.39	1.31–1.46	0.001
Grade (G3 vs. G1)	1.25	1.20–1.33	0.121	1.19	1.15–1.22	0.136
Menopausal status (postmenopausal vs. premenopausal)	1.19	1.13–1.31	0.138	1.21	1.13–1.26	0.141
BMI (≥25 vs. <25)	1.22	1.17–1.32	0.048	1.29	1.22–1.31	0.061
CRP level (≥5 vs. <5)	1.04	1.01–1.09	0.008	1.06	1.02–1.11	0.053
PON1 level (above vs. below median)	0.93	0.86–1.03	0.046	0.96	0.89–1.08	0.009

**Table 8:** Univariate and multivariate Cox regression model for PTX3 serum concentrations.

Univariate Analysis						
Variable	PFS			OS		
	HR	95% CI	*p*-Value	HR	95% CI	*p*-Value
Age (above vs. below median)	1.01	0.76–1.24	0.048	1.23	0.89–1.36	0.096
FIGO staging (III and IV vs. I and II)	1.62	1.29–1.76	0.013	1.54	1.37–1.65	0.021
Grade (G3 vs. G1)	1.24	1.06–1.39	0.022	1.34	1.27–1.58	0.133
Menopausal status (postmenopausal vs. premenopausal)	1.22	1.15–1.26	0.193	1.26	1.21–1.30	0.201
BMI (≥25 vs. <25)	1.31	1.28–1.33	0.087	1.13	1.05–1.19	0.116
CRP level (≥5 vs. <5)	1.18	1.11–1.24	0.059	1.11	1.07–1.15	0.043
PTX3 level (above vs. below median)	1.47	1.26–1.61	0.043	1.52	1.28–1.66	0.013
**Multivariate Analysis**						
	**PFS**			**OS**		
	**HR**	**95% CI**	***p*** **-Value**	**HR**	**95%CI**	***p*** **-Value**
Age (above vs. below median)	1.06	1.02–1.13	0.089	1.14	1.09–1.19	0.119
Stage (III and IV vs. I and II)	1.29	1.25–1.34	0.039	1.19	1.16–1.20	0.003
Grade (G3 vs. G1)	1.20	1.19–1.27	0.237	1.15	1.12–1.18	0.154
Menopausal status (postmenopausal vs. premenopausal)	1.07	1.03–1.10	0.113	1.01	0.94–1.07	0.262
BMI (≥25 vs. <25)	1.08	1.03–1.15	0.072	1.12	1.05–1.14	0.411
CRP level (≥5 vs. <5)	1.13	1.06–1.16	0.061	1.16	1.13–1.21	0.049
PTX3 level (above vs. below median)	1.24	1.21–1.28	0.134	1.31	1.22–1.37	0.321

## Data Availability

The data presented in this study are available on request from the corresponding author. The data are not publicly available due to ethical restrictions.
